# Development and Characterization of Spray-Dried Combined Levofloxacin–Ambroxol Dry Powder Inhaler Formulation

**DOI:** 10.3390/pharmaceutics16121506

**Published:** 2024-11-22

**Authors:** Ruwani K. Suraweera, Kirsten M. Spann, Emad L. Izake, Timothy J. Wells, Xiaodong Wang, Nazrul Islam

**Affiliations:** 1 Pharmacy Discipline, School of Clinical Sciences, Faculty of Health, Queensland University of Technology, Brisbane, QLD 4000, Australia; ruwanikaushalya.suraweera@hdr.qut.edu.au; 2 Centre for Immunology and Infection Control (CIIC), Faculty of Health, Queensland University of Technology, Brisbane, QLD 4000, Australia; kirsten.spann@qut.edu.au; 3 School of Chemistry and Physics, Faculty of Science, Queensland University of Technology, Brisbane, QLD 4000, Australia; e.kiriakous@qut.edu.au; 4 Frazer Institute, Faculty of Medicine, The University of Queensland, Brisbane, QLD 4102, Australia; timothy.wells@uq.edu.au; 5 Central Analytical Research Facility, Queensland University of Technology, Gardens Point Campus, Brisbane, QLD 4000, Australia; tony.wang@qut.edu.au

**Keywords:** levofloxacin, ambroxol, spray drying, pulmonary drug delivery, dry powder inhaler formulations

## Abstract

**Background:** This study explores the development and characterization of spray-dried composite microparticles consisting of levofloxacin (LVX, a broad-spectrum antibiotic), and ambroxol (AMB, a mucolytic agent that has antibacterial and antibiofilm properties), for the intended application of the drug against lower respiratory tract infections (LRTIs). **Methods:** A range of LVX to AMB mass ratios (1:1, 1:0.5, and 1:0.25) were prepared, with and without the use of the dispersibility enhancer leucine (LEU), and spray-dried following pre-optimized parameters to achieve the required particle size (1–5 µm) and flow properties. The formulations were characterized by attenuated total reflection-Fourier transform infrared (ATR-FTIR) spectroscopy, scanning electron microscopy (SEM), powder X-ray diffraction (PXRD), and a thermogravimetric analysis (TGA). The in vitro aerosolization performance of the new formulation was evaluated with a twin-stage impinger (TSI) at a flow rate of 60 ± 5 L/min. Using a validated RP-HPLC method, LVX and AMB were quantitatively determined. **Results:** The combined spray-dried LVX, AMB, and LEU particles were spherically shaped with sizes ranging from 1.9 to 2.9 µm, thus complying with the size requirements for effective deep lung deposition. The dispersibility enhancer leucine produced a high yield and enhanced the flow properties and aerosolization characteristics of the spray-dried formulations. The LVX to AMB mass ratios showed a remarkable impact on the aerosolization properties, with the LVX to AMB 1:1 mass ratio demonstrating the best flow and FPFs for both drugs. There must be a balanced ratio of these components for spray drying the composite particles to obtain composite particles of the required size and with the appropriate flow property. The addition of 5% of LEU significantly (*p* < 0.005) improved the FPF of all the formulations, probably by enhancing the surface hydrophobicity of the composite particles. **Conclusions**: The spray-dried combined antibiotics formulation has a strong potential for efficient lung delivery intended for the management of LRTIs.

## 1. Introduction

The increased recognition of pulmonary drug delivery as an effective and non-invasive treatment strategy against many chronic respiratory diseases, including lower respiratory tract infections (LRTIs), has led to a substantial interest in the development of inhaled dry powder formulations, particularly over the past twenty years [1]. Dry powder inhaler (DPI) formulations possess excellent solid-state stability and facilitate an efficient pulmonary delivery of drugs [2]. Further, dry powder formulations with combined antibacterials have proven to be beneficial in treating pulmonary infections, particularly those caused by biofilm-forming and antibiotic-resistant bacteria [3,4].

LRTIs have long been regarded as life-threatening and detrimental to both public health and the global economy [5]. The WHO recognized LRTIs as the foremost lethal communicable disease and the fourth primary contributing factor for global mortality, as illustrated by the 2.6 million deaths in 2019 [6]. Non-infectious disease conditions such as cystic fibrosis (CF), chronic obstructive pulmonary disease (COPD), and non-CF bronchiectasis (NCFB) are exacerbated by LRTIs [5]. Both gram-negative and gram-positive contagious bacteria such as *Streptococcus pneumonia*, *Pseudomonas aeruginosa*, and *Staphylococcus aureus* are the foremost causative bacterial pathogens of LRTIs [5].

LRTIs are commonly managed with oral or parenteral antibiotics [5,7,8,9]. Unfortunately, many commonly used systemic antibiotic formulations are not optimally effective at delivering sufficient concentrations of antibiotics to the infected lung cells to fully eradicate the causative pathogens [10,11]. As a result, systemic delivery often requires considerably elevated nominal doses to achieve the desired therapeutic levels of antibiotics at the targeted lung infection sites [12]. Consequently, long-term use of high-dose systemic antibiotics, particularly in patients with chronic lung diseases, has contributed to the development of antibiotic resistance, further complicating treatment efforts [9,11,13,14]. Inhalation drug delivery is an appealing alternative due to its promising benefits, such as an extensive lung surface area, high blood perfusion, higher peripheral epithelial permeability, low enzymatic activity, targeted pulmonary delivery, and the rapid onset of action [1]. Thus, compared to systemic antibiotics, inhaled antibiotics have demonstrated superior bactericidal activity and a lower propensity for the development of bacterial resistance due to site-specific delivery [15]. Further, inhaled antibiotics may eradicate resistant bacteria at a lower dose compared to systemic delivery [16,17,18].

Levofloxacin (LVX), a broad-spectrum synthetic fluoroquinolone, is effective against a wide range of gram-negative and gram-positive LRTI pathogens, including biofilm-forming bacteria [19,20,21,22]. The pulmonary delivery of LVX at significantly lower doses offers benefits in minimizing the dose-related systemic side effects by lowering the overall LVX dose requirement [23]. Combining LVX with ambroxol (AMB), a mucolytic agent, presents the unique therapeutic opportunities of synergism [24]. AMB, a repurposed drug, is further identified with notable individual antibacterial and antibiofilm activity [25,26] and particularly when combined with conventional antibiotics, such as vancomycin and ciprofloxacin [25,27,28]. Thus, when delivered together, AMB can potentially improve the localized LVX concentration in a LRT, enhancing the antibiotic penetrability [29] and demonstrating synergistic antibacterial activity [25,27,28]. Currently, the combination of LVX and AMB is available in tablet dosage form (500 mg LVX: 75 mg AMB) and is commonly prescribed for both upper respiratory tract infections (URTIs) and LRTIs [29,30]. However, the pulmonary delivery of the combined LVX and AMB might potentially reduce the associated systemic side effects of a tablet dosage form, and reduce the overall dose required while improving bioavailability.

Spray drying is a highly promising and easily up-scalable production technique for DPI formulations, offering a manipulation of the desired particle characteristics [23]. Dispersibility enhancers are commonly employed in DPI formulations to improve the flow and aerosolization properties. Leucine (LEU) is widely recognized as a safe and effective dispersibility enhancer in DPI formulations and is particularly well suited for spray drying process [31,32,33,34,35]. The in vitro cytotoxicity studies of DPI formulations with LEU reported the absence of cytotoxic effects when evaluated on lung epithelial cell lines (A549 and Calu-3) or alveolar cell lines (NR8383) [36,37,38]. Further, LEU’s ability to improve the deep-lung deposition of DPI formulations containing antibiotics, respiratory drugs, siRNA, and monoclonal antibodies is reported [38,39].

To date, very few studies have explored the DPI formulations of antibiotics combined with mucolytic agents. Lababidi et al. [40] investigated spray-dried formulations, including N-acetylcysteine and the antibiotics ciprofloxacin, tobramycin, and azithromycin. They produced a median mass aerodynamic diameter (MMAD) in the range of 2.16–2.63 µm, and a high fine particle fraction (FPF) above 60%. Lee et al. [27] developed a ternary DPI formulation (ciprofloxacin, gatifloxacin, AMB) including LEU with promising FPFs (64–69%). Furthermore, Guan et al. [41] developed ciprofloxacin nanocrystals and N-acetylcysteine co-spray-dried DPI formulations demonstrating %FPFs between 33.69 and 40.97%. In another study, the DPI formulations of antibiotics (LVX and ciprofloxacin) and mucolytic agents (N-acetylcysteine) were developed for the pulmonary infections associated with CF [4]. The spray-dried DPI formulations of an antibiotic (ciprofloxacin) with ternary components (quercetin and mucolytics such as N-acetylcysteine, mannitol, and AMB) against *Pseudomonas aeruginosa* biofilm infections in CF patients were investigated and demonstrated a high FPF (49%) [42]. Therefore, this study aims to develop and characterize the spray-dried inhaled composite particles resulting from combining LVX and AMB, with the dispersibility enhancer LEU, and their possible applications in the lung delivery of drugs against LRTIs.

## 2. Materials and Methods

### 2.1. Materials

Main active ingredients levofloxacin (LVX) (98.0–102.0% anhydrous basis- HPLC), ambroxol hydrochloride (AMB) (analytical standard) and excipient, leucine were purchased from Merck Life Sciences, Bayswater, VIC, Australia. All other chemicals and reagents used were analytical grade.

### 2.2. Optimization of Spray Drying Parameters

Spray-dried particles were prepared using a Buchi spray dryer (B-290, Buchi Labortechnik AG, Flawil, Switzerland). Preliminary experiments were conducted to optimize the spray-drying parameters for producing composite particles. LVX feed solutions were spray-dried with different parameters, i.e., inlet temperatures (Ti) of 100, 120, and 150 °C, gas flows of 357, 473, and 601 L/h, feed pump rates of 20, 25, and 30%, and feed concentrations of 0.5, 1, and 2 mg/mL. Then, the effect of the spray-drying parameters on the spray-drying yield and particle size of the resultant dried particles was determined. Based on these results, optimized spray-drying parameters were determined for further experiments.

### 2.3. Preparation of LVX and AMB Spray-Dried Formulations

Different mass ratios of LVX to AMB (Table 1) with or without incorporating 5% of LEU were dissolved in a 20:80 ratio of an ethanol–deionized water solvent system while maintaining a total feed solute concentration of 1 mg/mL. Based on the preliminary spray-drying studies, the constant spray-drying parameters used were as follows: T_i_ 120 ± 2 °C, aspiration was 100%, gas flow was 473 L/h, and feed pump rate was 20%, which lead to an outlet temperature (T_o_) between 60 and 80 °C. System stabilization was performed prior to each spray-drying process. The spray-dried formulations were placed in sealed vials and stored in a desiccator with silica gel at room temperature.

### 2.4. Particle Size and Morphology Analysis by Scanning Electron Microscopy (SEM)

The morphology of the raw LVX, AMB, and spray-dried particles was examined using SEM (Zeiss Sigma VP Field Emission SEM, Oberkochen, Germany). A thin layer of sufficient powder samples was applied on the aluminum sample stud covered with adhesive carbon tape. Loosely bound, excessive powder particles were removed from the adhesive carbon tape using pressurized nitrogen gas. To improve the conductivity of samples, mounted powder particles were coated with a 10 nm thick conductive gold film sputtered at 30 mA, and argon gas (pressure 0.5 mbar) for 75 s. Then, the coated powder particles were plasma cleaned for 1 min at 250 mT and imaged with the acquired high vacuum secondary electron images at an accelerating voltage of 3 kV, aperture size of 10 µm, and working distance of 8 mm. After acquiring SEM images, the particle size of spray-dried composites was measured by ImageJ software (v1.53e). Feret diameters of the 100 particles were measured resembling the physical particle size [43,44] and average particle sizes were calculated with OriginPro 2021 software.

### 2.5. Particle Density and Flow Property

Flow properties of the powder samples were determined with the powder flow parameters, Carr’s index (CI), Hausner ratio (HR), and angle of repose (θ), using Equations (1)–(3) [5].

(1) $$CL=\frac{100\;(V_{0}-V_{1})}{V_{0}}$$

(2) $$HR=\frac{\rho_{t}}{\rho_{b}}$$

(3) $$\theta =\tan^{-1}\left(\frac{2h}{d}\right)$$

Tapped density (ρ_t_) and bulk density (ρ_b_) of the powder formulations were calculated using Equations (4) and (5) [5].

(4) $$\rho_{b}=\frac{m_{0}}{V_{0}}$$

(5) $$\rho_{t}=\frac{m_{0}}{V_{1}}$$

The bulk density of the powder formulations was measured by filling 300 ± 0.5 mg (m_0_) of the formulation into a 5 mL graduated measuring cylinder without tapping. Then, the respective volume (V_0_) was recorded to calculate the ρ_b_. Subsequently, to measure the tapped density, the same powder-filled graduated measuring cylinder was fastened onto the tapped density tester (ERW-SVM101202, ERWEKA, Langen, Germany) and the final volume (V_1_) was recorded after carrying out 500 mechanical taps to calculate ρ_t_ [5]. Each powder formulation was tested in triplicates. The findings V_0_, V_1_, ρ_b_, and ρ_t_ were used to calculate CI and HR.

The angle of repose was calculated by measuring the angle from the cone-shaped powder pile to the horizontal plane [5]. An amount of 250 ± 0.5 mg of powder formulation was poured using gravity through a funnel (18 mm funnel mouth diameter, 33 mm height, 2 mm funnel end orifice diameter) into a flat weighing dish. The gap between the funnel end and the weighing dish was maintained at 3 cm. Then, the height (h) and diameter (d) of the powder pile were measured to calculate θ using Equation (3).

### 2.6. Attenuated Total Reflection- Fourier Transform Infrared (ATR-FTIR) Spectroscopy

The ATR-FTIR spectra of the raw LVX, AMB, and spray-dried powder samples were examined using an FTIR spectrophotometer (Thermo is5, Nicolet, Madison, WI, USA). A small amount of the powder sample was placed on the top of the diamond crystal and was secured with a high-pressure clamp. The ATR-FTIR spectrum was acquired using an 8 cm^−1^ resolution and 64 scans, and it was within the collection range of 4000–400 cm^−1^. A background scan was performed before each sample scan to minimize the interference of CO_2_ and water peaks with the FTIR spectrum. The acquired data and spectra were analyzed with OPUS analytical software (Bruker Alpha-P FTIR, Version 7.0 or 7.5, Bremen, Germany).

### 2.7. Thermogravimetric Analysis (TGA)

The decomposition and calorimetric behavior of the LVX, AMB, and spray-dried formulations were determined through a thermogravimetric analysis (NETZCCH Simultaneous Thermal Analyzer 449F3, Selb, Germany). Powder samples (2–3 mg) were placed in alumina sample cup, heated under nitrogen flux (80 mL/min) at the heating rate of 10 °C/min, and scanned from 30 to 500 °C. Then, the moisture content was calculated for each sample based on the weight loss percentages in relation to the temperature. An empty alumina sample cup was used to acquire the baseline for analysis.

### 2.8. Powder X-Ray Diffraction (PXRD)

The powder samples of LVX, AMB, LEU, F4, F6, and F8 were measured with capillary (internal diameter 1.0 mm) transmission geometry using a Rigaku SmartLab X-ray diffractometer (Rigaku, Tokyo, Japan). A focusing Goebel mirror in a CBO-E module was used to converge an X-ray beam from a Cu X-ray tube (λ = 1.54059 Å, 40 kV 40 mA), followed by the implementation of a height limiting slit of 15 mm. Soller slits of 2.5° were used on both the primary and secondary beam paths. A Hypix3000 detector (Rigaku, Tokyo, Japan) collecting diffraction signals in 1D mode with a PSD opening of 20 mm was also used, after an extended 6.6 mm anti-scattering slit and a 12 mm receiving slit were employed. The capillary samples were spun at 15 rpm during XRD pattern collection from 3 to 90° 2θ at a 0.02° step size in 1 h. The collected X-ray Diffraction data were compared with the ICDD PDF-5+ database using DIFFRAC.EVA v7 software. The Rietveld structural refinements were conducted with DIFFRAC.TOPAS V7 software (Figures S1–S3), in which the refined crystal structure and atomic positions were visualized in 3D. Further, according to the below Degree of Crystallinity (DOC) method (Equation (6)), the volume percentage of the crystalline part of the F8 was estimated.

(6) $$DOC=\frac{Area\;of\;the\;crystalline\;peaks}{\left(Area\;of\;the\;crystalline\;peaks\;+\;Area\;of\;Amorphous\;humps\right)}\;\times \;100\%$$

Note the contributions from capillary glass have been excluded from the above calculation.

### 2.9. Drug Analysis by Reverse Phase High Performance Liquid Chromatography (RP-HPLC)

The determination of LVX and AMB was performed using an in-house developed RP-HPLC method with UV detection using an Agilent HPLC Series 1100 with a Column heater and Fluorescence Detector (Heracles, Hewlett–Packard, Waldbronn, Germany). The isocratic mobile phase composition was 72:28 with a pH 3 phosphate buffer: acetonitrile and Agilent Poroshell 120 EC-C18 (4.6 × 250 mm, 4 μm) column with a 4 µm guard column (Agilent, Santa Clara, CA, USA) was used for the stationary phase. The HPLC operational parameters were as follows: the injection volume was 20 µL, flow rate was 1 mL/min, run time was 10 min, and detection wavelength was 220 nm. Deionized water was used as the sample preparation solvent and the stock solutions were sonicated for 2 min to ensure the complete dissolution of LVX and AMB. OpenLAB CDS ChemStation edition C.01.10 (201) was used to operate the HPLC.

#### 2.9.1. Mobile Phase Preparation

To prepare the pH 3 phosphate buffer solution, 3 g of potassium dihydrogen orthophosphate was dissolved in 1 L of deionized water in a volumetric flask and the pH was adjusted with 80% *w*/*v* phosphoric acid. Then, the buffer solution was vacuum-filtered through a 0.45 µm membrane filter [45]. To prepare the mobile phase, the pH 3 phosphate buffer solution and acetonitrile were mixed well in a 72:28 volume ratio and degassed for 15 min in an ultrasonic bath.

#### 2.9.2. Method Validation

Method validation of the developed HPLC method was performed according to a modified procedure followed by Maharini et al. [45] in accordance with the International Harmonization Conference (ICH) guidelines. As an internal standard, 50 µg/mL of the LVX and AMB solutions were prepared by diluting 1 mg/mL of the solution. The standard was run in three replicates to determine the system suitability of the developed HPLC method and the HPLC spectrum parameters (peak area, retention time, tailing factor, resolution, and theoretical plates) were statistically analyzed for reproducibility. The specificity of the HPLC method was determined by comparing the spectra of the LVX-AMB formulations with and without the excipient LEU. The linearity of the developed method was determined by developing calibration curves for LVX and AMB with a dilution series of the 1 mg/mL standard (0.1, 0.25, 0.5, 1, 5, 10, 15, 20, 25, 30, 50, and 100 µg/mL). To determine the accuracy, three known concentrations (5, 20, and 50 µg/mL) of LVX-AMB were run in triplicates and compared with the concentration values obtained from the calibration curves. Intra-day precision was examined by running the 5, 20, and 50 µg/mL LVX-AMB samples for 3 days and statistically analyzing the standard deviation. To examine inter-day precision, the 5, 20, and 50 µg/mL LVX-AMB samples were analyzed 2 times on day 1. All the samples were run in triplicate.

### 2.10. In Vitro Aerosolization by Twin-Stage Impinger (TSI)

The in vitro aerosolization performance of the spray-dried powder formulations was determined using a twin-stage impinger (TSI, Apparatus A; British Pharmacopoeia, 2000) following the recommended British Pharmacopeial method [5]. First, stage 1 and stage 2 of the TSI were filled with 7 mL and 30 mL of the solvent (deionized water), respectively. After securing the TSI tightly with clamps, an air flow rate of 60 ± 5 L/min was applied at the mouthpiece of the TSI with a vacuum pump (D-63150, Erweka, Heusenstamm, Germany) and monitored with a digital flow meter (Model 10A3567SAX, Fisher and Porter, Telford, UK). After stabilizing the air flow rate at 60 ± 5 L/min, the vacuum pump was switched off. Then, a Size 3 hard gelatin capsule (Vcaps^@^ Plus, Capsugel, Lonza, Basel, Switzerland) was filled with the spray-dried powder formulation (20 ± 1 mg) and inserted into the inhaler device (Breezehaler^®^, Novartis Pharmaceuticals Pvt Ltd., Macquarie Park, NSW, Australia). Then, the breezehaler was connected tightly to the mouthpiece of the TSI apparatus. The capsule was pierced with the aid of the inhaler device. Then, the powder particles were aerosolized at a 60 ± 5 L/min air flow rate for 5 s to allow powder flow through the TSI apparatus and deposition at different stages. Powder particles deposited in the device, stage 1 (S1) and stage 2 (S2) were separately washed with deionized water and collected in 100 mL volumetric flasks. The volumetric flasks were sonicated for 2 min to ensure the complete dissolution of particles, and the drugs were quantified with the previously described, validated RP-HPLC method. Emitted dose (ED) and FPF with regards to the ED and recovered dose (RD) of the drugs were determined by HPLC and calculated following Equations (7)–(9), where S1, S2, and RD are stage 1 of TSI, stage 2 of TSI, and RD, respectively. RD is defined as the total amount of drug collected from S1, S2, and the inhaler. Each formulation was analyzed in five separate capsules and the average was calculated.

(7) $$ED=\frac{\left(S1+S2\right)}{RD}\times 100$$

(8) $$FPF(RD)=\frac{S2}{RD}\times 100$$

(9) $$FPF\left(ED\right)=\frac{S2}{ED}\times 100$$

### 2.11. Statistical Analysis

A statistical analysis was performed by applying a one-way analysis of variance (ANOVA) and Tukey’s post hoc test. A *p* value < 0.05 was considered as a significant difference.

## 3. Results and Discussion

### 3.1. Optimization of Spray Drying Parameters

The yields and the size of the resulting spray-dried particles under various conditions are presented in Table 2. The yield of the spray-dried formulations ranged from 16.8 to 34.4%. A notable observation was that a substantial proportion of the particles adhered to the chamber walls of the spray dryer rather than reaching the collection vessel, leading to low yields. This has also been observed in other studies [42]. However, the inclusion of LEU increased the spray-drying yield by reducing adhesion to the chamber walls. The spray-drying yield decreased with an increasing T_i_ and gas flow rate, while it was improved by an increased feed solid concentration. No distinct trend was observed with the changing feed pump rates. Moreover, system stabilization followed by a 15 min water run and increased feed volumes improved the spray-drying yield (Table 2). The particle size increased with a high feed pump rate, a reduced gas flow rate, and an increased feed concentration. However, the particle sizes remained within the inhalable range (1–5 µm) for all the formulations [46].

### 3.2. Preparation of Spray-Dried Formulations

The varying mass ratios of LVX to AMB were selected as 1:1, 1:0.5, and 1:0.25 to optimize the formulation regarding the desirable physicochemical, micromeritic, and therapeutic characteristics (Table 1). The solutions of LVX and AMB, with or without LEU, were spray-dried, and the corresponding yields and particle sizes were determined (Table 3). The yield of the spray-dried formulations was between 40.8 and 55.3%. As evidenced by the formulations F4, F6, and F8, when compared with the formulations without LEU, the incorporation of 5% of LEU significantly (*p* < 0.005) improved the spray-drying yield. Further, formulation F4, which contained the highest percentage of AMB with LEU, demonstrated the highest yield at 55.3%. As the physicochemical and micromeritic characteristics of the spray-dried particles have a significant impact on the aerosolization properties [46], parameters such as particle size, particle morphology, FTIR, TGA, XRD, flow properties, and aerodynamic parameters were evaluated to ensure the optimization of the formulations.

### 3.3. Particle Size

**Figure 1:**
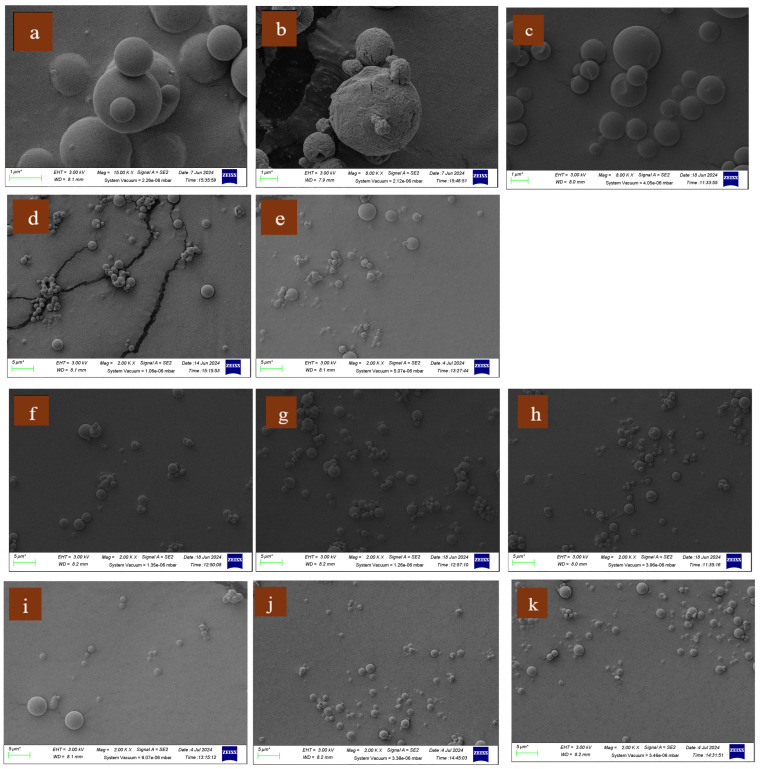
Scanning electron microscopy images of F2(*15K) (**a**), F1(*8K) (**b**), F3(*8K) (**c**), F2(*2K) (**d**), F1(*2K) (**e**), F3(*2K) (**f**), F4(*2K) (**i**), F5 (*2K) (**g**), F6(*2K) (**j**), F7 (*2K) (**h**), and F8 (*2K) (**k**).

The particle size of the spray-dried formulations was analyzed using SEM, as reported in Figure 1. Among the spray-dried particles, the largest particle size was exhibited by F1 (2.9 ± 1.3 µm) and the smallest by F4 (1.9 ± 1.2 µm). Despite this variation, all the formulations fell within the particle size range of 1.9 to 2.9 µm, complying with the appropriate particle size for effective deep lung deposition, which is 1 to 5 µm [46,47]. Notably, the inclusion of LEU led to a reduction in particle size across the spray-dried formulations (Table 3); however, a statistically significant decrease (*p* < 0.05) was observed only in the LVX (1:1) formulations. Similar findings were observed in LVX co-spray-dried particles with theophylline and LEU [48]. Amphiphilic LEU possesses the ability to reduce the surface tension of an aqueous spray-drying feed due to its surfactant-like properties. Thus, upon the atomization of a feed solution, smaller droplets are generated resulting in smaller spray-dried powder particles [34]. In the spray-dried formulations without LEU, a trend in declining particle size was observed with the increasing percentage of AMB, from 20% to 50%, as evidenced by F3, F5, and F7. However, a statistically significant reduction (*p* < 0.05) in particle size compared to F1 was observed only upon the addition of 50% AMB.

### 3.4. Particle Morphology

Figure 1 displays the SEM images of the spray-dried individual and combination formulations of LVX and AMB. All the formulations exhibited a spherical geometry (1.9 to 2.9 µm diameter), which is considered a favorable morphological feature for inhalation drug delivery. The spherical particles exhibit smooth flow behavior due to their ability to move with minimal resistance, facilitated by a gliding motion [49]. Although spherical particles typically present with higher aerodynamic diameters compared to rod/needle-shaped particles, they were reported to have high FPF values [47]. As supported by the literature, the particles likely aggregated during the spray-drying process in the absence of a dispersing agent such as LEU [32]. In the presence of LEU, most of the particles in the spray-dried formulations were non-aggregated and only minor particle aggregations were observed, of which the overall aggregate diameters were predominantly below 5 µm (Figure 1). This mostly non-aggregated state may enable the efficient aerosolization of the spray-dried particles [46]. As reported in previous studies, LEU enhances aerosolization and flow properties by preventing particle aggregation [48,50].

### 3.5. Powder Density and Flowability

The powder density and flowability parameters of the spray-dried composite particles were determined and presented in Table 3. The bulk density of all the spray-dried formulations ranged from 0.18 to 0.30 g/cm^3^. The particles with a bulk density of less than 0.4 g/cm^3^ are considered optimal for easy dispersion and deep lung deposition upon aerosolization [47]. An inverse relationship between particle size and bulk density was observed among the spray-dried formulations. Larger particles tend to exhibit lower bulk densities due to the increased presence of voids between the particles [51]. However, such a trend was not observed in the spray-dried formulations with 5% of LEU. Moreover, the bulk density of the spray-dried formulations significantly (*p* < 0.05) increased upon the addition of both LEU and AMB.

The flow properties of the spray-dried formulations were further evaluated in terms of CI, HR, and angle repose (Table 3). The flow of the formulations was interpreted based on the flow parameters and limits published by Ain et al. [52]. The results implied that the poor flow properties of F1 were improved with the addition of AMB and LEU. Upon the inclusion of AMB, the flow property improvement was statistically significant (*p* < 0.05) with regards to θ, but statistically insignificant with the other flow parameters, namely CI and HR (*p* > 0.05). Similarly, the flow properties of all the spray-dried LVX and AMB combination formulations were improved with the addition of 5% of LEU. Even small amounts of flow-enhancing excipients have been shown to significantly improve the flow characteristics of the formulations [49]. In this study, a statistically significant (*p* < 0.05) improvement in θ was observed across all the formulations with varying LVX:AMB mass ratios upon the addition of LEU. However, statistically significant improvements in CI and HR (*p* < 0.05) were observed only in the formulations with an LVX to AMB ratio of 1:0.25. The anti-adhesive properties of LEU are responsible for its dispersibility enhancing ability which could improve flow properties [32,33,53]. Among the formulations, F4 exhibited the best flow properties, suggesting that F4 has the potential to improve and maintain consistent powder handling, loading, and discharge from an inhaler device, as well as an efficient aerosolization upon inhalation [47].

### 3.6. Fourier Transform Infrared (FTIR) Spectroscopy

**Figure 2:**
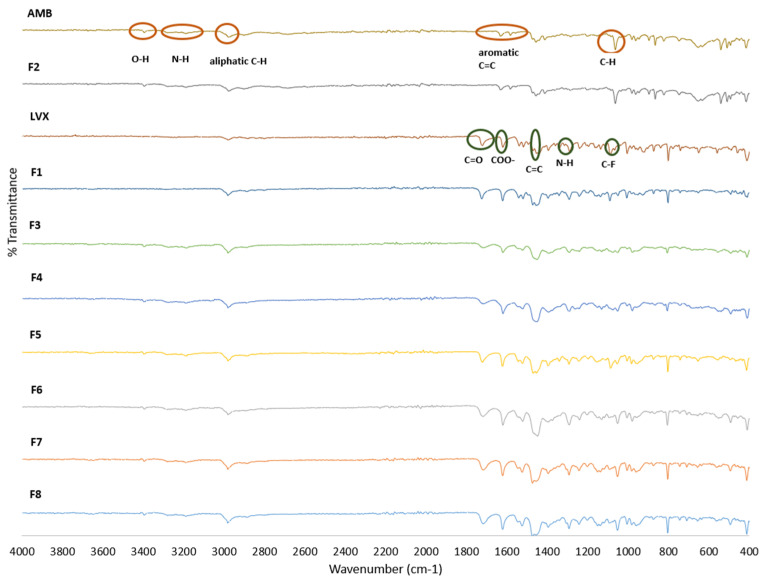
FTIR spectra of levofloxacin, ambroxol, and spray-dried formulations.

The FTIR spectra were collected from the raw and spray-dried formulations to indicate any potential drug–drug and drug–excipient interactions (Figure 2). The FTIR spectra of LVX, AMB, and the spray-dried formulations were comparable to those reported in the literature [42,54,55,56,57]. The characteristic bands of AMB (Figure 2; AMB) were observed at 3280 cm^−1^ and 3229 cm^−1^ and correspond to the N–H stretching vibration of the aromatic amine and N–H stretching vibration of the aliphatic amine, respectively. An aromantic C–H stretching vibration band was also observed at 3185 cm^−1^. These characteristic bands were observed in the spray-dried AMB formulation (Figure 2; F2) as well. However, the N-H bands were broad and demonstrated minor shifts upon spray drying (F3 to F8). Similar results were obtained by Alhajj et al. and the shift was attributed to the conversion of crystalline AMB to its amorphous form [42]. The band observed at 3393 cm^−1^ is associated with the O–H group and was observed in AMB, F2, and the spray-dried combination LVX:AMB formulations. The bands at 1064 cm^−1^ and 1068 cm^−1^ are attributable to the stretching vibrations of C–H in AMB and F1. The bands observed at 1630–1411 cm^−1^ are associated with aromatic C=C vibrations and were retained in all the spray-dried LVX:AMB formulations [55], but overlayed with the LVX bands in the combination formulations.

Characteristic bands were observed in LVX (Figure 2; LVX and F1) at 1724 cm^−1^ (stretching vibration of C=O), 1620 cm^−1^ (deprotonated COO^−^), 1449 cm^−1^ (C=C aromatic bond in the quinolone structure), 1291 cm^−1^ (N–H stretching), and 1049 cm^−1^ (C–F bond) [56,57]. In the spray-dried LVX:AMB formulations (F3 to F8), the vibration bands of the carbonyl/carboxyl groups, amine stretching, and C-F were observed in the ranges of 1720–1724 cm^−1^, 1618–1620 cm^−1^, 1440–1447 cm^−1^, 1290–1291 cm^−1^, and 1049–1051 cm^−1^, respectively. The characteristic band intensities declined with a decreasing LVX ratio in the formulations. Of interest, the characteristic peaks of both the raw LVX and AMB were retained in the spray-dried formulations without major shifts, indicating the absence of drug–drug or drug–excipient interactions in the prepared formulations.

### 3.7. Thermogravimetric Analysis (TGA)

**Figure 3:**
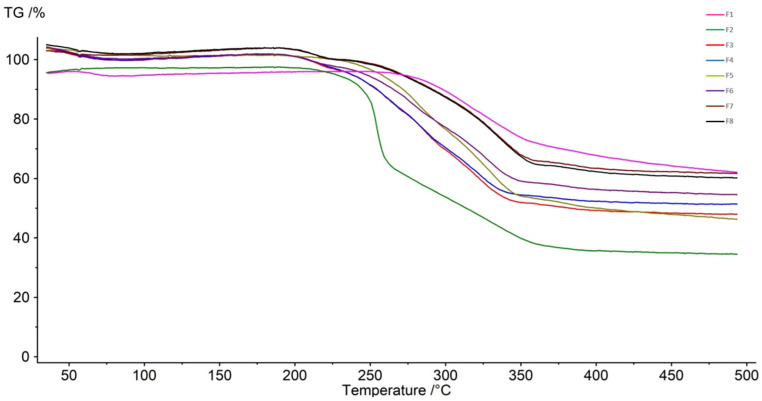
TGA thermograms of levofloxacin (LVX), ambroxol (AMB), and spray-dried LVX:AMB combination formulations (F3–F8).

The thermal behavior of LVX, AMB, and the spray-dried LVX:AMB combination formulations (F3-F8) were investigated with TGA. The TG curve of LVX appeared as two-phase mass losses. Initially, at around 57 °C, 2.38% mass loss was observed due to water desorption (Figure 3), which aligns well with the theoretical water content of Levofloxacin hemihydrate (LVXH) (2.43%) [58]. No further substantial mass losses were observed until the major mass loss occurred at 257 °C due to the decomposition of LVX [58]. In the TG curve of AMB (Figure 3), two-stage mass loss started around 200 °C, which is attributed to the decomposition of AMB [59,60]. Furthermore, all the developed spray-dried formulations (F3-F8) demonstrated the major thermal behavior of both LVX and AMB. However, the spray-dried LVX:AMB formulations tended to decompose before pure LVX, at around 200 °C, due to the incorporation of AMB. Therefore, according to the TG curves, all of the formulations were thermally stable up to 200 °C.

### 3.8. Powder X-Ray Diffraction (PXRD)

#### 3.8.1. Crystal Structure of API Component Phases

The qualitative phase analysis results suggest that the three component phases (Table 1) used in this experiment are as follows: (1) LVXH (PDF# 00-065-1246); (2) ambroxol hydrochloride (PDF# 00-069-1516) (AMBHCL); and (3) L-Leucine (PDF# 02-063-2332) (Table 4).

#### 3.8.2. XRD Phase Analysis of the Product Samples

**Figure 4:**
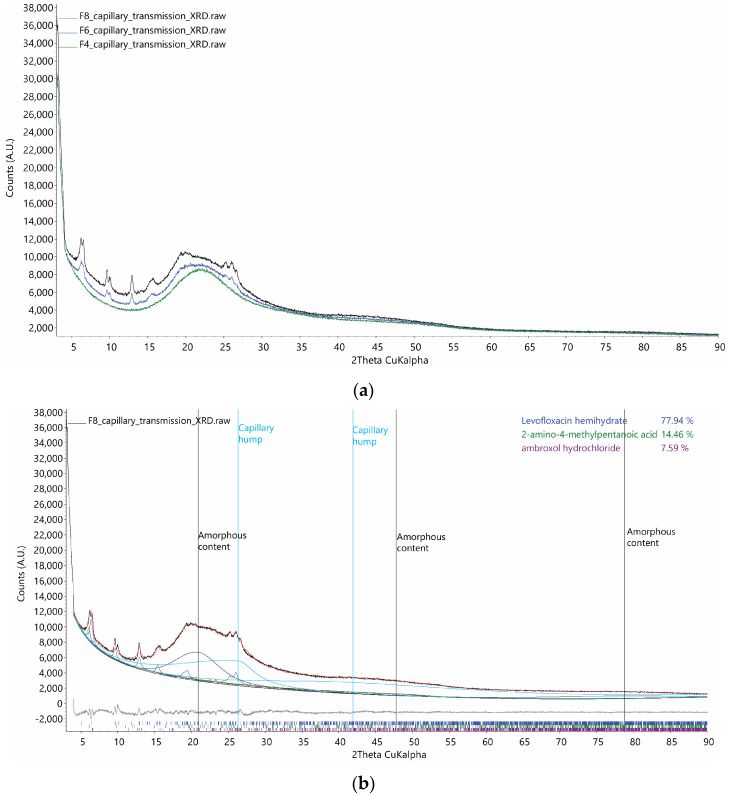
(**a**) XRD patterns collected for samples F4, F6, and F8. (**b**) Rietveld quantitative phase analysis of sample F8. Relative weight ratios among the re-crystallized phases are shown in the top right corner. Fitting of amorphous contents using split-pseudo-Voigt peaks are indicated using vertical lines. The two capillary humps shapes are fixed according to the fitting of the fully crystalline levofloxacin XRD data.

It can be seen from Figure 4a that samples F4, F6, and F8 are mainly amorphous as most of the X-ray scattering forms broad humps. Sample F4 is completely amorphous with no sharp crystalline peak detected, while samples F6 and F8 show minor sharp crystalline peaks. The intensity of the crystalline peaks increases with the LVX content (Table 1). It can be inferred that the residual water in the spray-drying process (Table 3) can cause the LVX molecules to re-crystallize in the LVXH phase. Previous DSC studies reported similar observations in their differential scanning calorimetry results where the distinct endothermic peak associated with LVX was retained in all spray-dried LVX formulations. This can be attributed to the partial crystallization of the amorphous LVX, due to a dehydration process [61].

To confirm this hypothesis, the crystal structure of LVXH and the other source phases were used to fit the minor crystalline peaks of sample F8, which shows the highest crystalline peaks (Figure 4b). The major intensity contributions are from the amorphous content in formulation F8, which are fitted by three asymmetric peaks (vertical lines labelled as “Amorphous content” in Figure 4b). From the relative weight percentage of the re-crystallized phases, LVXH is the main re-crystallized phase, though most of the molecules are still in an amorphous form. According to the DOC method, the volume percentage of the crystalline part of F8 is estimated to be 29.4%.

### 3.9. Drug Analysis by RP-HPLC

The simultaneous determination of LVX and AMB was successfully achieved with the in-house developed RP-HPLC method. The phosphate buffer (pH 3): acetonitrile (72:28 *v*/*v*), was used in the mobile phase for isocratic elusion. Method validation was performed by assessing the system’s suitability, specificity, linearity, accuracy, and precision. The measured parameters of the developed HPLC method (the retention time, area, tailing factor, resolution, and number of theoretical plates) met the criteria of acceptance for both LVX and AMB [45]. In terms of specificity, the absence of additional peaks or peak shifts upon the addition of 5% of LEU indicates the high selectivity of the developed HPLC method towards LVX and AMB (Figure S4). Linear standard curves were obtained for LVX and AMB, within the concentration range of 5–100 µg/mL and 1–100 µg/mL, respectively. The linear regression equations for LVX and AMB were y = 44.016x + 553.83 (R^2^ = 0.9989) and y = 52.248x + 308.73 (R^2^ = 0.9920), respectively. The limit of detection (LOD) was 3.66 µg/mL for LVX and 3.25 µg/mL for AMB. Further, the limit of quantification (LOQ) was 11.10 µg/mL for LVX and 9.86 µg/mL for AMB. The accuracy of the method, evaluated at concentrations of 5, 20, and 50 µg/mL, met the % recovery criteria (80–110%) [45] and was in the range of 98.3–99.6% for LVX and 98.5–101.3% for AMB. The intra-day and inter-day precision study results [with a relative standard deviation (RSD) between 0.1 and 2.0%] revealed that the developed HPLC method has an acceptance value of RSD < 2, according to Maharini et al. [45].

### 3.10. In Vitro Aerosolization Performance

The aerosolization properties of the spray-dried formulations were evaluated in terms of the % of RD, % of ED, % FPF(RD), and % FPF(ED) as presented in Table 5. The % of RD for both LVX and AMB across all the spray-dried formulations ranged from 93.9% to 100.6%, indicating an efficient particle dispersion from the device and deposition in different stages of the TSI. Both LVX and AMB exhibited relatively similar aerosolization properties (FPF) from the ED across all the individual formulations.

The data demonstrate a clear correlation between the LVX to AMB ratio and the aerosolization performance of the spray-dried formulations. For instance, the formulations with a 1:1 LVX to AMB ratio exhibited a superior FPF(RD) (Table 5; F3 (39.0% for LVX, 37.5% for AMB), F4 (48.7% for LVX, 48.3% for AMB), F9 (52.8% for LVX, 50.1% for AMB), and F10 (58.1% for LVX and 53.6% for AMB) and superior ED values compared to the formulations with a lower AMB content, including the 1:0.5 and 1:0.25 ratio formulations. This indicates that a reduction in AMB may adversely affect the aerosolization efficiency of the spray-dried formulations. Thus, there must be a balanced ratio of these components for spray drying the composite particles. A similar trend was observed with flow properties, which could be attributed to the observed particle size reduction upon the inclusion of AMB in the formulations (Table 3).

Similarly, the incorporation of LEU improved the aerosolization performance of the formulations, resembling the trend observed in the flow property parameters. In particular, the increment in %FPF for both LVX and AMB, with regards to both RD and ED, was statistically significant (*p* < 0.005) with the addition of 5% of LEU in the formulations (F4, F6, F8), compared to the respective formulations without LEU (F3, F5, F7). This could be due to the relatively low CI, HR, and θ, which contribute to better flowability [42]. Furthermore, the increased LEU concentration further enhanced the aerosolization performance of the spray-dried combination LVX and AMB formulations, with the formulation containing 10% leucine (F10, with a 1:1 LVX to AMB ratio) achieving the highest FPF (RD) of 58.1% for LVX and 53.6% for AMB. However, the increments in %FPF observed upon changing the %LEU from 5% to 7.5% and 7.5% to 10% were not statistically significant (*p* > 0.05). Therefore, a 5% LEU was selected as the optimized LEU concentration for this study. Further, excessive LEU content might result in the decreased stability of the formulations [32]. Accordingly, F4 presented with the best %FPF (RD) for both LVX (48.7%) and AMB (48.3%). However, with regards to ED, F8 presented with the best %FPF for both LVX (68.2%) and AMB (69%). LEU is an amphiphilic amino acid that exhibits anti-adhesive properties, which contribute to an enhanced flowability, and an improved aerosolization [32,33,34]. The ability of LEU to minimize particle cohesion and promote better powder dispersibility likely accounts for the enhanced aerosolization performance [32,62,63]. Additionally, as both LVX and AMB are hydrophilic compounds, amphiphilic LEU is expected to orient on the surface of spray-dried particles, with the hydroxyl groups orienting towards the drug particles and the hydrophobic domains orienting towards the surface of the composites. Hence, the surface hydrophobicity of the spray-dried composites reduces the particle interactions leading to enhanced flowability and dispersibility [32,33,62]. It also possessed the ability to modify the surface morphology and density of the powder formulations upon drying, further optimizing their performance [35,62,63]. The fine particle dose (FPD) representing the amount of LVX and AMB deposited in the S2 of the TSI following the aerosolization of a 20 mg capsule ranged from 3.9 ± 0.2 mg to 7.2 ± 0.5 mg for LVX, and from 1.3 ± 0.1 mg to 4.8 ± 0.2 mg for AMB. It is expected that these amounts will be deposited in the deep lungs following the inhalation of a single capsule [5]. Nevertheless, in vivo pharmacokinetic studies are necessary to determine the therapeutic pulmonary concentrations of the inhaled combination LVX and AMB formulations. For all the spray-dried formulations, the FDP mass ratios between LVX and AMB recovered from the S2 of the TSI were in good agreement with their nominal mass ratios in the formulations. This suggested the absence of substantial particle segregation during the formulation handling, aerosolization, and deposition processes [42].

## 4. Conclusions

We demonstrated a feasible and effective spray-drying approach to develop LVX/AMB composite microparticles that have the appropriate particle size, flow, and aerosolization properties and are suitable for developing DPI formulations. The parameters of the spray-drying method substantially impacted the yield and the particle size of the formulations. The prepared LVX/AMB/LEU formulations demonstrated spherical-shaped particles between 1.9 and 2.9 µm, thus allowing for effective deep lung deposition. FTIR confirmed the compatibility of LVX, AMB, and LEU in the formulations. The inclusion of LEU significantly improved the yield, particle size, flow properties, and aerosolization characteristics of the new spray-dried LVX/AMB formulations. The statistically significant (*p*< 0.005) enhancement of the FPF for both LVX and AMB was observed upon the addition of 5% LEU to the formulations. Overall, the findings of the research demonstrate the importance of controlling the operating parameters of the spray-drying process and the significance of utilizing an efficient dispersibility enhancer, such as LEU, for the development of inhalable composite particles with enhanced flow properties. These findings provided new insights into the optimum parameters required to synthesize efficient inhalable formulations of LVX/AMB composite microparticles for the deep lung delivery of drugs against LRTIs. Further in vitro and in vivo studies are warranted to evaluate the therapeutic efficacy and pulmonary concentrations of LVX and AMB in these inhaled composite formulations.

## Figures and Tables

**Table 1:** Composition of spray-dried formulations.

Formulation Code	Mass Ratio (LVX: AMB)	LVX (%*w*/*w*)	AMB (%*w*/*w*)	LEU (%*w*/*w*)
F1	1:0	100	0	0
F2	0:1	0	100	0
F3	1:1	50	50	0
F4	1:1	47.5	47.5	5
F5	1:0.5	66.6	33.3	0
F6	1:0.5	63.3	31.7	5
F7	1:0.25	80	20	0
F8	1:0.25	76	19	5
F9	1:1	46.25	46.25	7.5
F10	1:1	45	45	10
F11	1:0.5	61.7	30.83	7.5
F12	1:0.5	60	30	10
F13	1:0.25	74	18.5	7.5
F14	1:0.25	72	18	10

Note: SD—spray-dried, F1: SD-LVX, F2: SD-AMB, F3: SD-LVXAMB(1:1), F4: SD-LVXAMB(1:1)_5%LEU, F5: SD-LVXAMB(1:0.5), F6: SD-LVXAMB(1:0.5)_5%LEU, F7: SD-LVXAMB(1:0.25), F8: SD-LVXAMB(1:0.25)_5%LEU, F9: SD-LVXAMB(1:1)_7.5%LEU, F10: SD-LVXAMB(1:1)_10%LEU, F11: SD-LVXAMB(1:0.5)_7.5%LEU, F12: SD-LVXAMB(1:0.5)_10%LEU, F13: SD-LVXAMB(1:0.25)_7.5%LEU, F14: SD-LVXAMB(1:0.25)_10%LEU.

**Table 2:** Effect of the spray-drying operational parameters on the yield product.

Formulation	Inlet Temperature (°C)	Pump Rate (%)	Gas Flow Rate (L/h)	Feed Concentration (mg/mL)	Particle Size (µm)		Yield (%)		Outlet Temperature (°C)	
1 *	100	25	473	1	2.62		31	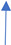	55–65	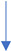
2 *	120	25	473	1	3.01		24.3		65–68	
3 *	150	25	473	1	2.71		22		82–93	
4 *	120	20	473	1	2.57	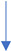	33.6		71–74	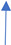
2 *	120	25	473	1	3.01		24.2		65–68	
5 *	120	30	473	1	3.18		29.2		62–65	
6 *	120	25	357	1	3.64	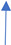	24.8	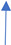	65	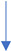
2 *	120	25	473	1	3.01		24.2		65–68	
7 *	120	25	601	1	2.43		18.4		67–68	
8 *	120	25	473	0.5	2.79	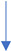	16.8	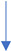	50–60	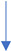
2 *	120	25	473	1	3.01		24.2		65–68	
9 *	120	25	473	2	3.95		33.5		67–70	
10 *	120	25	473	1	2.99		31.6		69–72	
11 **	120	25	473	1	2.78		34.4		69–72	

* Feed volume 50 mL. ** Feed volume 125 mL. Formulation 10* represents formulation 2* repeated with stabilization for 15 min with water. Formulation 11** represents formulation 2* repeated with a higher feed volume.

**Table 3:** Yield, particle size, and flow properties of spray-dried formulations (mean ± SD, *n* = 3).

Formulation Code	Yield (%)	Moisture Content (%)	Particle Size (µm)	Bulk Density (g/mL)	Tap Density (g/mL)	Car’s Index	Hausner’s Ratio	Angle of Repose	Flow
F1	40.8	2.38	2.9 ± 1.2	0.18 ± 0.01	0.27 ± 0.01	31.56 ± 3.59	1.46 ± 0.08	45.89 ± 0.44	Poor
F2	45.9	0	2.2 ± 1.3						
F3	46.7	3.03	2.5 ± 1.3	0.30 ± 0.02	0.38 ± 0.00	19.87 ± 4.01	1.25 ± 0.06	38.84 ± 1.67	Fair
F4	55.3	4.01	1.9 ± 1.2	0.25 ± 0.01	0.29 ± 0.02	15.11 ± 2.69	1.18 ± 0.04	34.47 ± 1.34	Good
F5	45.2	1.65	2.6 ± 1.7	0.23 ± 0.01	0.29 ± 0.02	22.34 ± 6.62	1.29 ± 0.11	42.58 ± 0.71	Passable
F6	49.0	3.79	2.5 ± 1.1	0.27 ± 0.03	0.34 ± 0.04	18.28 ± 1.67	1.22 ± 0.03	35.03 ± 1.35	Good
F7	42.5	1.28	2.7 ± 1.1	0.20 ± 0.00	0.27 ± 0.00	27.24 ± 0.99	1.37 ± 0.02	44.04 ± 0.83	Passable
F8	43.8	3.16	2.3 ± 1.4	0.21 ± 0.00	0.27 ± 0.01	21.18 ± 0.43	1.27 ± 0.01	40.29 ± 0.34	Passable

**Table 4:** Crystallographic parameters from Rietveld structural refinement of their XRD data.

Component Phases		Levofloxacin Hemihydrate	Ambroxol Hydrochloride	L-Leucine
Chemical Formula		C18 H21 F N3 O4.5	C13 H19 Br2 Cl N2 O	C6 H13 N O2
Space Group		C121	C2/c	P21
Lattice Parameters	*a* (Å)	29.1478(7)	25.2952(8)	9.6269(3)
	*b* (Å)	6.88913(4)	15.5517(5)	5.3277(2)
	*c* (Å)	18.8591(5)	8.1947(3)	14.6669(3)
	*α* (°)	90	90	90
	*β* (°)	114.086(3)	94.354(3)	93.963(4)
	*γ* (°)	90	90	90
Unit Cell Volume (Å^3^)		3457.1(1)	3214.4(2)	750.45(4)
Molecules No. in Cell Z		8	8	4

**Table 5:** Aerosolization results of spray-dried levofloxacin and ambroxol formulations (mean ± SD, *n* = 5).

	LVX					AMB				
Formulation Code	RD (%)	ED (%)	FPF(%) from RD	FPF(%) from ED	FPD (mg) from RD	RD (%)	ED (%)	FPF(%) from RD	FPF(%) from ED	FPD(mg) from RD
F1	93.9 ± 3.1	55.3 ± 1.8	26.3 ± 1.0	47.6 ± 0.8	5.3 ± 0.2	-	-	-		
F3	98.2 ± 2.2	67.6 ± 3.4	39.0 ± 1.9	57.8 ± 1.1	3.9 ± 0.2	97.4 ± 3.1	66.8 ± 3.4	37.5 ± 2.2	56.1 ± 0.9	3.8 ± 0.2
F4	98.0 ± 3.1	72.9 ± 4.3	48.7 ± 4.0	66.7 ± 2.8	4.7 ± 0.4	98.3 ± 4.1	72.8 ± 2.7	48.3 ± 1.6	66.3 ± 1.1	4.6 ± 0.2
F5	97.1 ± 4.6	60.3 ± 2.6	34.3 ± 1.5	56.8 ± 0.9	4.6 ± 0.2	96.2 ± 5.5	60.2 ± 4.4	33.7 ± 2.7	55.9 ± 1.5	2.3 ± 0.2
F6	98.0 ± 4.9	69. 5 ± 2.7	44.8 ± 1.5	64.5 ± 1.0	5.7 ± 0.2	96.7 ± 5.8	69.5 ± 4.3	44.4 ±2.6	63.8 ± 1.7	2.8 ± 0.2
F7	96.4 ± 3.9	54.4 ± 2.2	30.8 ± 1.3	56.6 ± 0.8	4.9 ± 0.2	94.4 ± 4.1	55.4 ± 3.9	31.7 ± 4.7	57.2 ± 3	1.3 ± 0.1
F8	97.8 ± 4.1	61.9 ± 2.2	42.1 ± 1.3	68.2 ± 1.1	6.4 ± 0.2	95.1 ± 4.6	63.1 ± 2.8	43.4 ± 4.4	69.0 ± 4.3	1.7 ± 0.2
F9	97.9 ± 3.2	73.9 ± 4.4	52.8 ± 4.2	71.4 ± 2.8	4.9 ± 0.4	99.4 ± 2.8	73.9 ± 3.5	50.1 ± 2.7	67.7 ± 1.7	4.6 ± 0.3
F10	98.2 ± 3.2	74.2 ± 4.5	58.1 ± 4.3	78.2 ± 3.0	5.2 ± 0.3	101.0 ± 4.4	77.9 ± 4.4	53.6 ± 2.3	68.8 ± 1.6	4.8 ± 0.2
F11	97.0 ± 3.5	69.7 ± 3.3	48.9 ± 2.3	70.3 ± 4.9	6.0 ± 0.3	99.0 ± 4.8	68.8 ± 6.7	45.2 ± 4.3	65.5 ± 3.7	2.8 ± 0.4
F12	99.2 ± 3.7	70.1 ± 3.5	49.6 ± 2.2	70.8 ± 4.7	5.9 ± 0.3	99.1 ± 2.8	70.3 ± 8.0	47.1 ± 5.1	66.7 ± 4.1	2.8 ± 0.5
F13	98.7 ± 4.2	64.4 ± 3.4	45.8 ± 3.3	71.3 ± 3.6	6.7 ± 0.4	100.6 ± 1.9	63.8 ± 1.8	45.2 ± 4.6	71.4 ± 2.5	1.7 ± 0.2
F14	99.2 ± 3.4	65.4 ± 3.4	50.1 ± 3.7	76.9 ± 2.3	7.2 ± 0.5	100.3 ± 2.9	65.8 ± 5.9	47.7 ± 1.3	72.2 ± 3.6	1.7 ± 0.4

## Data Availability

All the relevant data are available in this article.

## Supplementary Materials

The following supporting information can be downloaded at https://www.mdpi.com/article/10.3390/pharmaceutics16121506/s1, Figure S1: (a) Refinement of Levofloxacin hemihydrate molecule structure in unit cell according to XRD pattern; and (b) Refined crystal structure of Levofloxacin hemihydrate; Figure S2: (a) Refinement of ambroxol hydrochloride molecule structure in unit cell according to XRD pattern; and (b) Refined crystal structure of ambroxol hydrochloride; Figure S3: (a) Refinement of L-Leucine molecule structure in unit cell according to XRD pattern; and (b) Refined crystal structure of L-Leucine; Figure S4: HPLC spectrums of 50 µg/mL of levofloxacin and ambroxol standard with (a) and without (b) 5% of leucine; and Table S1: Results of HPLC system suitability test of levofloxacin and ambroxol in the mobile phase with pH 3 phosphate buffer: acetonitrile (72:28 *v*/*v*%).
