# Supplementary material for: Development and Characterization of Spray-Dried Combined Levofloxacin–Ambroxol Dry Powder Inhaler Formulation
Source: Pharmaceutics. 2024 Nov 22;16(12):1506. doi: 10.3390/pharmaceutics16121506 (PMC11728515; doi:10.3390/pharmaceutics16121506)
Supplement: Supplementary file 1 [file pharmaceutics-16-01506-s001.zip › pharmaceutics-3307453-supplementary.pdf]

## Supplementary Data

a)

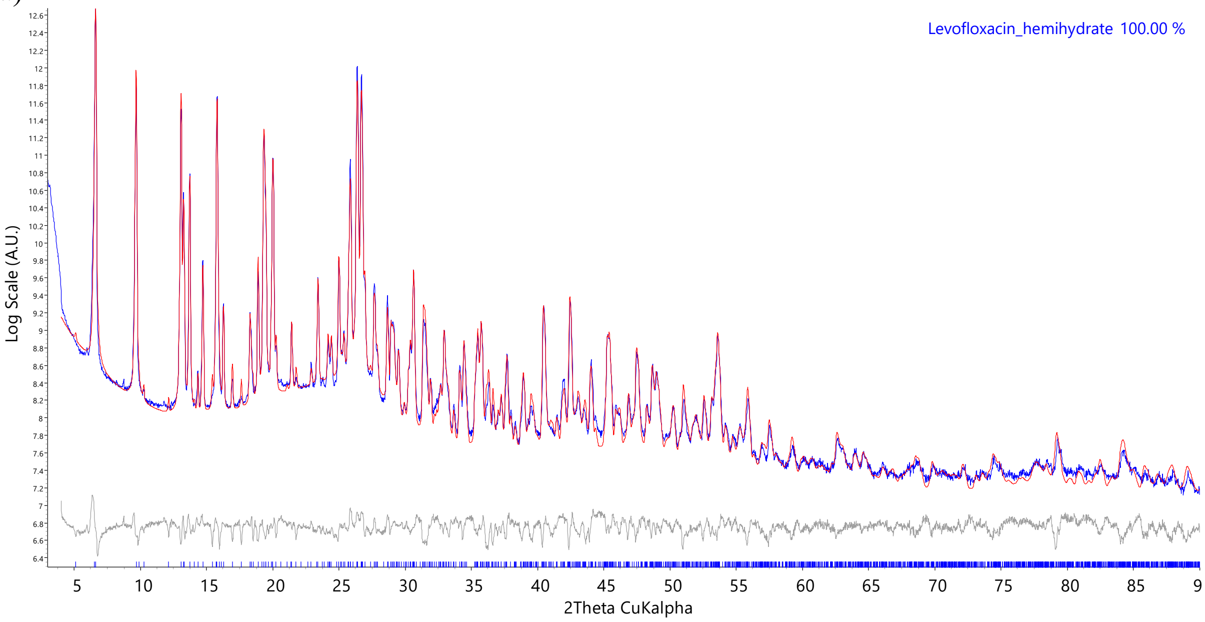


b)

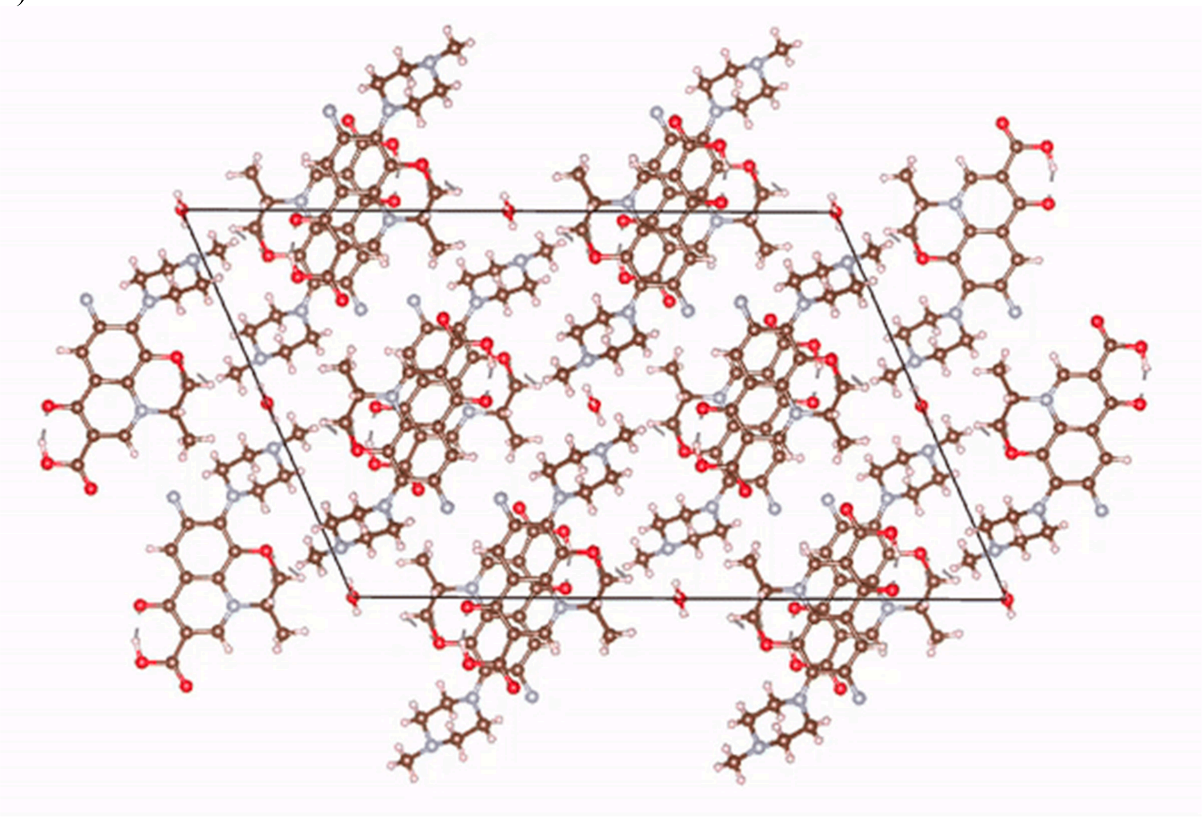

Figure S1. a) Refinement of Levofloxacin hemihydrate molecule structure in unit cell according to XRD pattern; b) Refined crystal structure of Levofloxacin hemihydrate.

a)

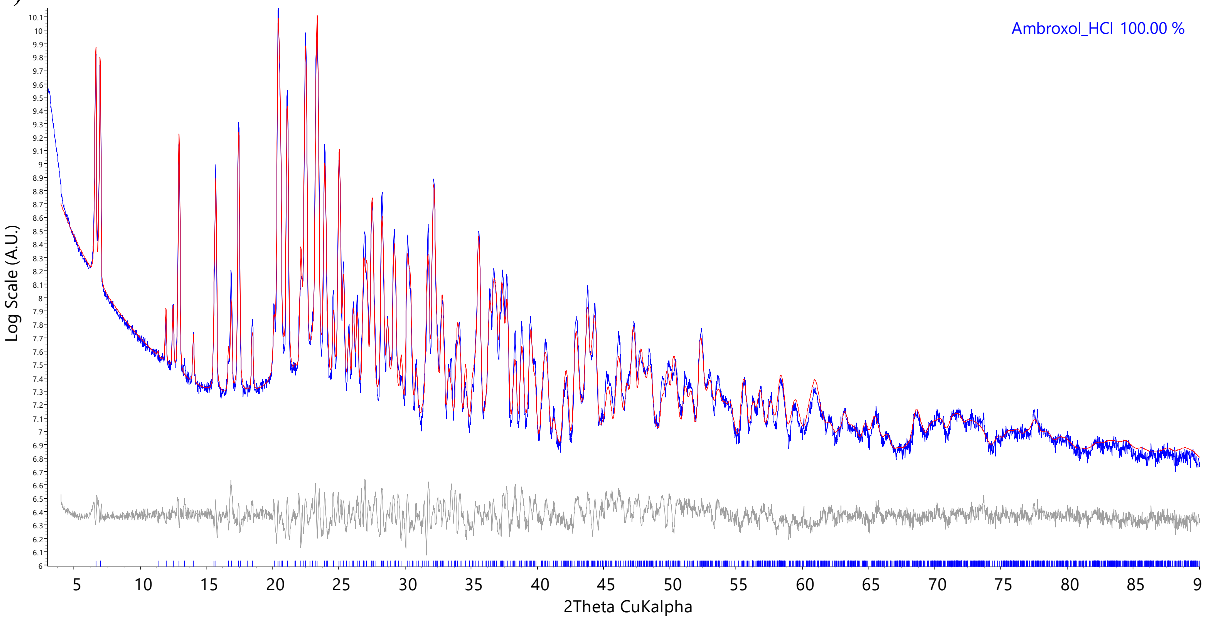


b)

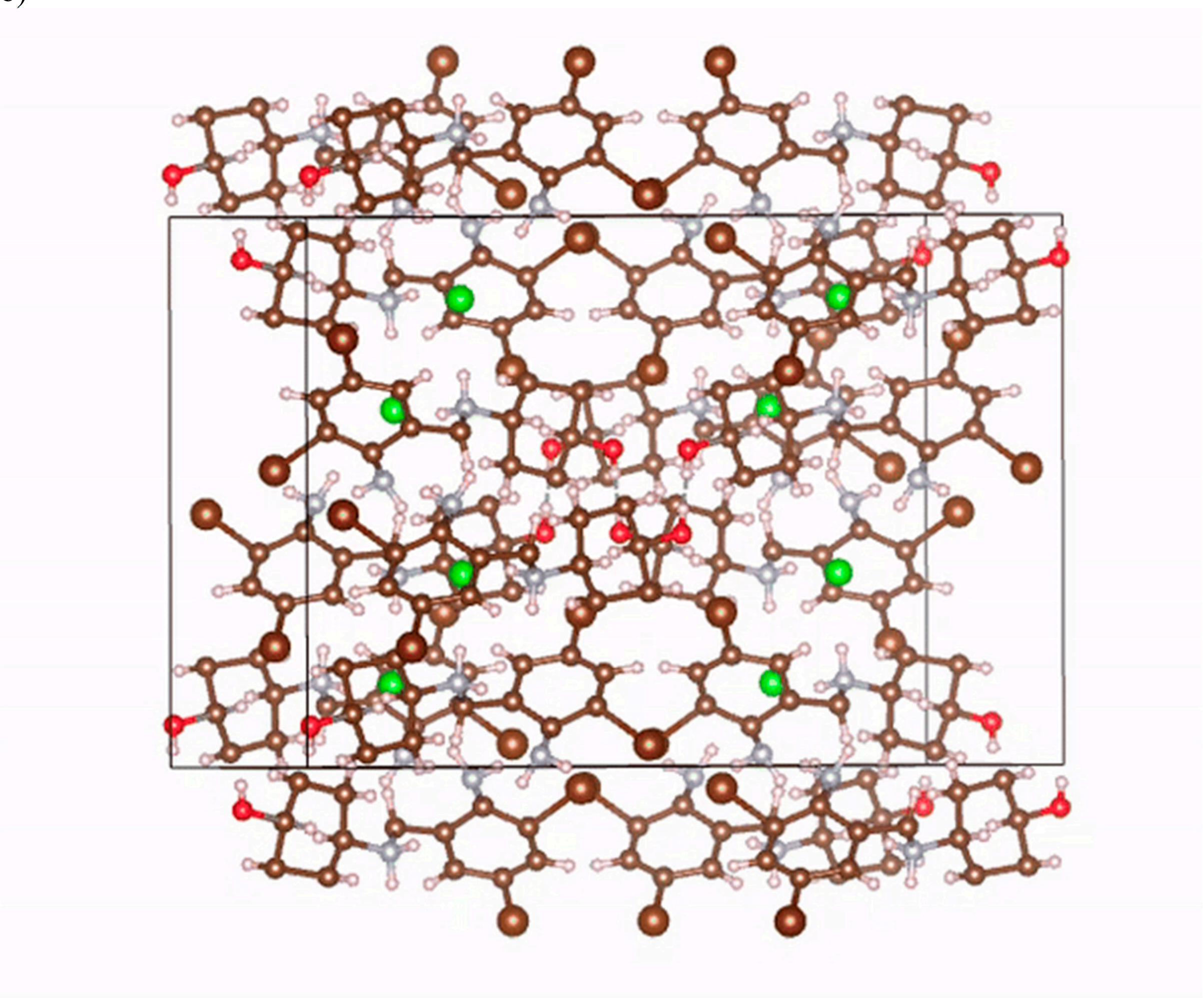

Figure S2. a) Refinement of Ambroxol hydrochloride molecule structure in unit cell according to XRD pattern; b) Refined crystal structure of Ambroxol hydrochloride.

a)

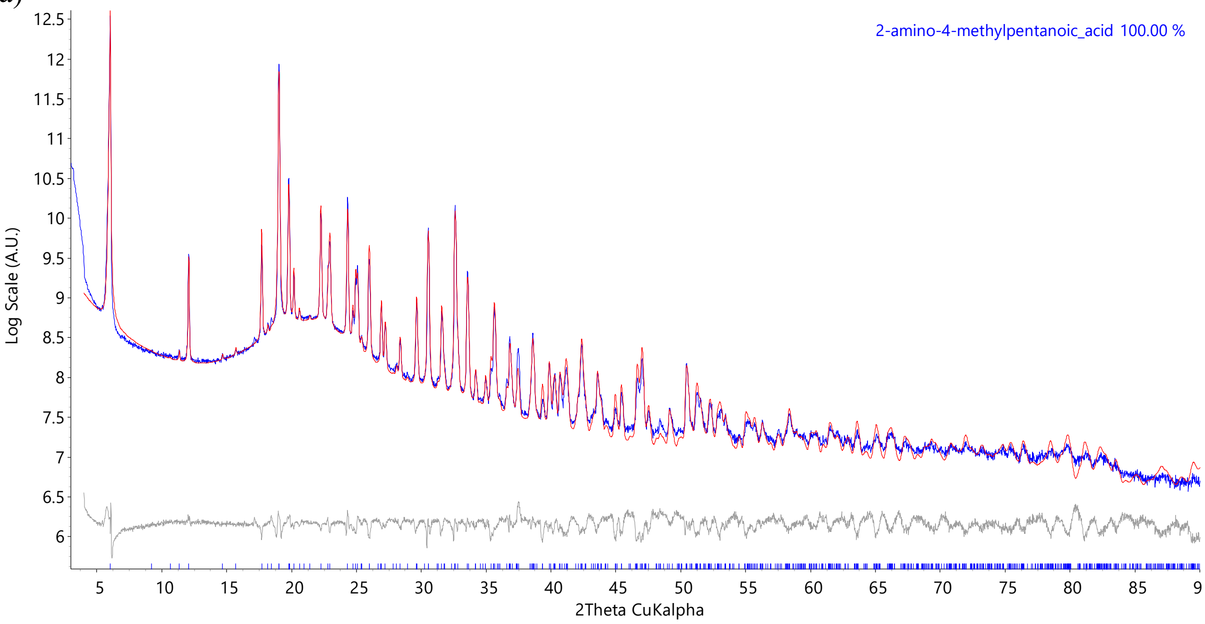


b)

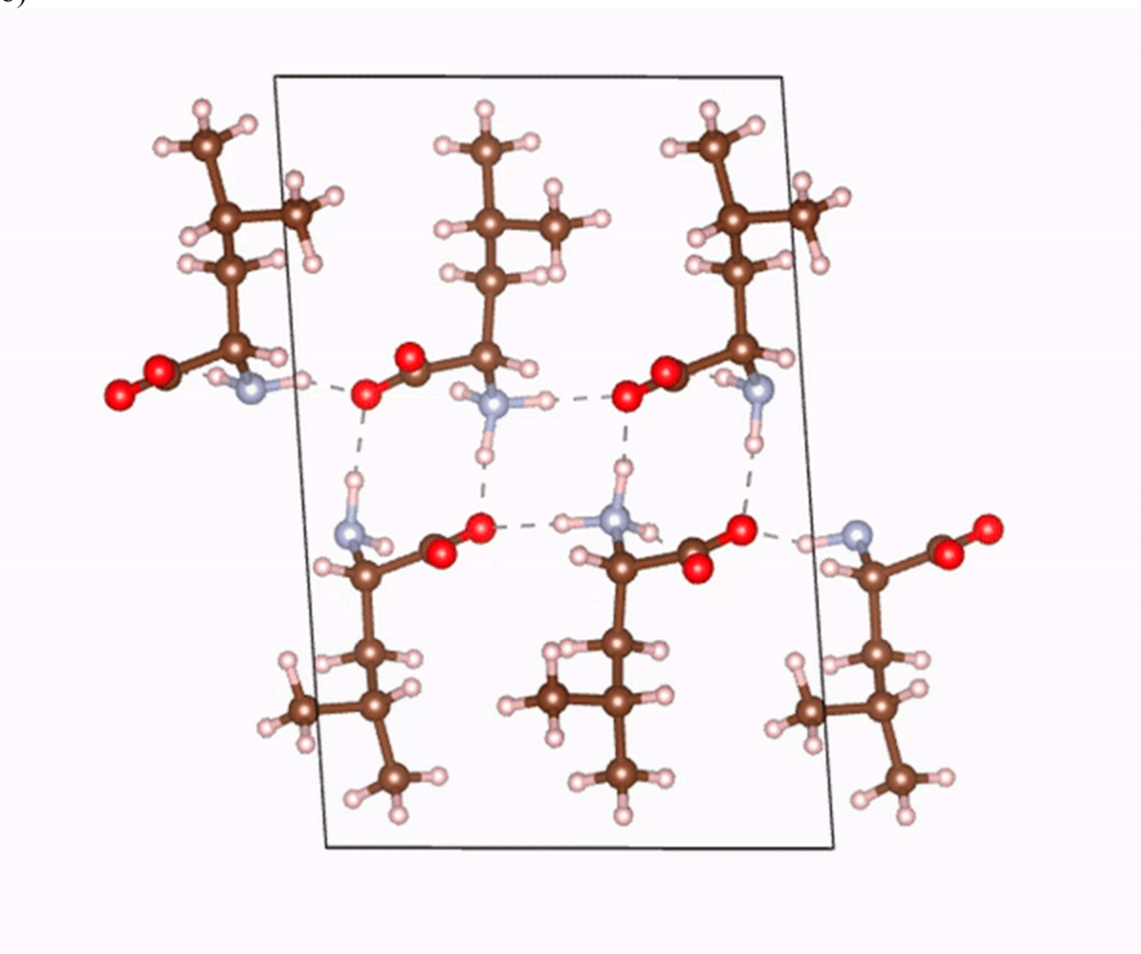

Figure S3. a) Refinement of L-Leucine molecule structure in unit cell according to XRD pattern; b) Refined crystal structure of L-Leucine.

Table S1: Results of system suitability test of levofloxacin and ambroxol HPLC system in the mobile phase pH 3 phosphate buffer: acetonitrile (72:28 v/v%)

| **Parameters** | **LVX Average (n=3)** | **RSD (%)** | **AMB Average (n=3)** | **RSD (%)** | **Criteria of acceptance** |
|---|---|---|---|---|---|
| Retention Time (minutes) | 3.15 | 1.01 | 5.55 | 0.69 | RSD <2% |
| Area | 2760.50 | 1.88 | 2798.19 | 1.23 | RSD <2% |
| Tailing Factor (TF) | 1.15 | 1.05 | 0.98 | 1.82 | TF <2 |
| Resolution (RS) | 13.03 | 0.81 | 13.03 | 0.81 | RS >2 |
| Number of Theoretical Plates (N) | 9142.20 | 3.51 | 8856.38 | 0.42 | N >2000 |

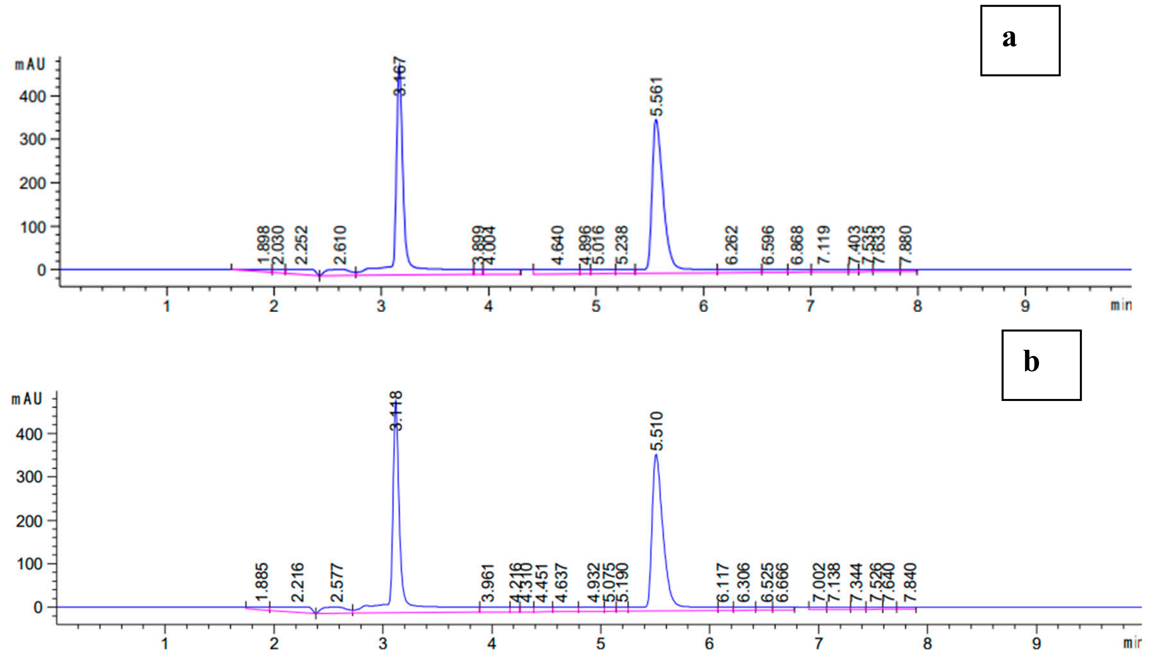


Figure S4: HPLC spectrums of 50 µg/mL levofloxacin and ambroxol standard with [(a)] and without [(b)] 5% of leucine
